# Effect of closing the internal ring during laparoscopic TAPP for Nyhus type III indirect inguinal hernia on postoperative seroma: a retrospective cohort study

**DOI:** 10.3389/fsurg.2026.1897447

**Published:** 2026-07-29

**Authors:** Dan Li, Hui Zhang, Hui Wang, Xuan Zeng, Long Chen

**Affiliations:** Department of General Surgery, Guangdong Provincial People's Hospital, Zhuhai Hospital (Jinwan Central Hospital of Zhuhai), Zhuhai, Guangdong, China

**Keywords:** complications, laparoscopic surgery, seroma, TAPP, type III indirect inguinal hernia

## Abstract

**Objective:**

To investigate whether sutured closure of the internal ring during laparoscopic transabdominal preperitoneal patch plasty (TAPP) reduces postoperative seroma formation in patients with Nyhus type III indirect inguinal hernia.

**Methods:**

We retrospectively analyzed clinical data from 266 male patients who underwent surgery for unilateral primary Nyhus type III indirect inguinal hernia between January 2022 and December 2025. Patients were stratified into an experimental group (*n* = 133, intraoperative internal ring closure) and a control group (*n* = 133, no internal ring closure) according to whether surgeons performed internal ring suturing during operation. Baseline demographics, maximum internal ring defect diameter, 24 h postoperative Visual Analogue Scale (VAS) pain score, operative duration, intraoperative blood loss, length of postoperative hospital stay, seroma incidence, hernia recurrence rate, and other complications were compared between the two cohorts.

**Results:**

Baseline characteristics were balanced between the two groups (all *P* *>* *0.05*). Operative duration was significantly longer in the experimental group compared with the control group [(56.84 ± 7.80) min vs. (54.66 ± 7.88) min, *P* *=* 0.02]. No significant intergroup differences were detected in intraoperative blood loss, 24 h postoperative VAS score, or postoperative hospital stay (all *P* *>* *0.05*). The incidence of postoperative seroma was markedly lower in the experimental group than in the control group [2.30% (3/133) vs. 9.02% (12/133), *P* *=* 0.03]. Two hernia recurrences were observed exclusively in the control group [1.50% (2/133) vs. 0.00% (0/133), *P* *=* 0.50]; however, the short follow-up duration precluded any definitive conclusions about intergroup differences in recurrence risk. No other severe complications occurred in either group.

**Conclusion:**

Intraoperative internal ring closure is a safe and feasible adjunct to TAPP for Nyhus type III indirect inguinal hernia, only slightly prolonging operative duration. This technique lowers the risk of early postoperative seroma, yet long-term recurrence outcomes remain to be validated in larger cohorts with extended follow-up.

## 1 Introduction

Laparoscopic inguinal hernia repair, including transabdominal preperitoneal patch plasty (TAPP) and totally extraperitoneal (TEP) repair, offers advantages over conventional open surgery, such as reduced trauma, less postoperative pain, and shorter hospital stays, and has become one of the primary surgical approaches for adult inguinal hernia repair. Despite a relatively low overall complication rate, postoperative seroma remains one of the most common complications in clinical practice (1). The incidence of seroma following TAPP is approximately 3%–8%, typically occurring within the first week after surgery (2). Although most seromas will naturally absorb within three months after the surgery, larger seromas may cause local pain, affect wound healing, and in severe cases, lead to secondary infection. Such complicated seromas may require percutaneous aspiration, prolong clinical recovery, and raise medical expenses (3). Therefore, effective strategies to reduce seroma risk carry substantial clinical value.

Previous studies have confirmed that seroma formation is closely associated with multiple risk factors, such as hernia sac size, the scope of intraoperative tissue dissection, thermal tissue trauma induced by ultrasonic shears and other energy devices, and residual dead space after hernia repair (4). This clinical issue is especially prominent in patients with Nyhus type III indirect inguinal hernia, defined as defects ≥3 cm or hernia contents extending into the scrotum. The large hernia sac leaves extensive preperitoneal dead space after dissection, which predisposes patients to fluid accumulation and seroma development. Multiple surgical interventions have been proposed to mitigate seroma, including hernia sac transection, sac fenestration, preperitoneal closed-suction drainage, and scrotal elevation. However, research findings remain inconsistent; several of these methods add operative complexity or raise infection risks (5–8).

In laparoscopic direct inguinal hernia repair, robust clinical evidence indicates that sutured closure of the direct hernia defect (e.g., plicating the pseudo-sac or fixing it to Cooper's ligament to eliminate dead space) significantly reduces postoperative seroma and recurrence rates (9–11). A subsequent meta-analysis further verified that closing the direct defect during laparoscopic repair lowers seroma risk without increasing surgical complications (12). Nevertheless, few studies have explored whether internal ring closure delivers comparable benefits for indirect inguinal hernias, and high-quality conclusive evidence is absent. Indirect and direct inguinal hernias differ substantially in anatomical architecture, hernia sac course, and preperitoneal dissection range; therefore, surgical strategies validated for direct hernias cannot be directly applied to indirect lesions.

To fill this research gap, the present retrospective cohort study compared the impact of intraoperative internal ring closure vs. standard non-closure TAPP on postoperative seroma incidence in patients with Nyhus type III indirect inguinal hernia. We also evaluated secondary endpoints including operative duration, intraoperative blood loss, postoperative pain, hospital stay, and recurrence rate, aiming to provide an optimized surgical strategy for routine clinical practice.

## 2 Materials and methods

### 2.1 Study design and ethics approval

This single-center retrospective cohort study was approved by the Institutional Ethics Committee of our hospital (Ethics Approval No.: 2026057H). All patients provided written informed consent before TAPP surgery. The institutional review board waived the requirement for separate informed consent for retrospective follow-up data collection.

### 2.2 Study population

We enrolled adult male patients who received surgical treatment at our hospital between January 1, 2022 and December 31, 2025. All patients had a preoperative diagnosis of unilateral primary Nyhus type III indirect inguinal hernia confirmed intraoperatively (maximum hernia defect diameter ≥3 cm or hernia contents extending into the scrotum). All participants underwent complete TAPP without intraoperative placement of preperitoneal drainage tubes.

### 2.3 Inclusion criteria

(1) Age ≥18 years, male;
(2) Intraoperatively confirmed as Nyhus type III unilateral primary indirect inguinal hernia;
(3) First inguinal hernia repair;
(4) Underwent complete TAPP procedure;
(5) Postoperative follow-up duration ≥6 months with complete follow-up data.

### 2.4 Exclusion criteria

(1) Recurrent hernia;
(2) Inability to tolerate general anesthesia due to major organ dysfunction (e.g., cardiac or pulmonary insufficiency);
(3) History of lower abdominal or inguinal region surgery;
(4) Preoperatively or intraoperatively confirmed as incarcerated or strangulated hernia that could not be reduced;
(5) Conversion to open surgery during the procedure for any reason;
(6) Preoperative long-term use of anticoagulant or antiplatelet agents;
(7) Surgery date less than 6 months prior, loss to follow-up within 6 months, or missing clinical data.

### 2.5 Grouping method

Electronic medical records and surgical operation notes were retrieved to stratify patients into two groups based on whether surgeons performed sutured closure of the internal ring intraoperatively: an experimental group (internal ring closure) and a control group (conventional TAPP without internal ring closure).

### 2.6 Surgical technique

All operations were performed by a single senior surgeon with extensive experience in laparoscopic inguinal hernia repair. Mesh products, mesh fixation techniques, and suture materials were standardized across both groups. The implant used was the Shanshi-Xi PASL mesh (National Medical Products Administration Approval No.: 20173130899), and sutures consisted of 3-0 absorbable barbed sutures (Aipu, BD31172AS-5). Routine preperitoneal closed-suction drainage was not placed in any patient.

**Figure 1:**
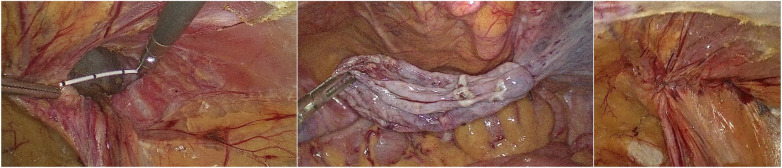
Surgical steps for internal ring closure. **(1)** Measurement of the internal ring defect diameter; **(2)** ligation and dissection of the hernia sac; **(3)** sutured closure of the internal ring.

All patients received standardized TAPP under general anesthesia following official laparoscopic inguinal hernia surgery guidelines. The operation process adopted by the experimental group is shown in Figure 1. The inner ring closure technique is as follows

(1) Suture placement: Continuous suturing was performed from the lateral to medial margin of the internal ring using a reverse stitching technique with gradual tensioning to obliterate the enlarged internal ring orifice.
(2) Suture layers: The inferior stitch plane targeted the iliopubic tract; surgeons took care to avoid deep needle penetration to prevent injury to the iliohypogastric, ilioinguinal, and genitofemoral nerves. The superior stitch plane incorporated the conjoint tendon and transversalis fascia.
(3) Spermatic cord protection: As suturing advanced toward the medial border of the internal ring, the spermatic cord vessels were gently retracted medially to fully expose the underlying iliopubic tract and transversalis fascia before needle insertion, avoiding accidental suturing or entrapment of the spermatic cord.
(4) Maintenance of cord patency: After suture tightening, the internal ring was adjusted to allow smooth passage of dissecting forceps tips, maintaining a patent channel for the spermatic cord to prevent vascular stricture or compression-induced ischemia.

## 3 Observation indicators

### 3.1 Baseline demographic data

Patient age, height, weight, BMI, and intraoperative laparoscopic measurement of the maximum internal ring defect diameter.

### 3.2 Perioperative indicators

(1) Operative time: from skin incision to wound closure (min);
(2) Intraoperative blood loss (mL);
(3) Postoperative 24 h pain score: assessed using the Visual Analogue Scale (VAS, 0–10 points);
(4) Postoperative hospital stay (days).

### 3.3 Follow-up and complications

#### 3.3.1 Seroma

Definition and diagnostic criteria:

Seroma was defined as palpable or ultrasonographically confirmed fluid collection in the surgical field. Diagnosis required both criteria below:

(1) Postoperative soft tissue swelling localized to the inguinal or scrotal region;
(2) Either palpable fluctuation on clinical examination or ultrasonographic identification of an anechoic fluid collection within the subcutaneous or preperitoneal plane.

**Classification criteria:**

The classification criteria for seroma are based on the standards proposed by Morales-Conde S (13): Type I: Clinically palpable seroma without ultrasonographic fluid collection;

- Type II: Ultrasonographically confirmed and clinically palpable seroma;
- Type III: Symptomatic seroma requiring percutaneous aspiration intervention;
- Type IV: Secondarily infected seroma requiring additional clinical management.

Recorded data included the time of initial seroma detection, duration of spontaneous resolution, any interventional procedures, and final clinical outcome.

#### 3.3.2 Hernia Recurrence

Recurrence was defined as re-protrusion of hernia contents through the inguinal hernia ring, confirmed by physical examination or inguinal ultrasound at any follow-up visit.

#### 3.3.3 Other complications

Wound infection, postoperative hemorrhage, chronic inguinal pain (persisting >3 months postoperatively), spermatic cord entrapment, and iatrogenic nerve injury.

### 3.4 Follow-up method

Post-discharge follow-up was conducted via outpatient consultation, telephone interviews, and online communication tools (WeChat, QQ). Follow-up began on the date of surgery, with a minimum required follow-up duration of 6 months. Standardized follow-up time points included postoperative day 3, week 1, month 1, month 3, and month 6, with extended follow-up scheduled beyond 6 months. Patients presenting with seroma, persistent pain, or abnormal sensory symptoms were instructed to return for weekly outpatient review; clinicians documented symptom duration, interventions administered, and final recovery status.

### 3.5 Statistical analysis

Statistical analyses were performed using SPSS 21.0 software. Continuous variables are presented as mean ± standard deviation and compared between groups via independent-samples t-tests. Categorical variables are reported as count (percentage) and compared using the chi-square test; Fisher's exact test was applied when expected cell frequencies were less than 5. All statistical analyses were two-tailed; *P* *<* 0.05 was defined as the threshold for statistical significance.

## 4 Results

### 4.1 Baseline patient characteristics

A total of 266 patients were enrolled in this study, with 133 patients in the experimental group and 133 patients in the control group.

As shown in Table 1 baseline demographic and anatomical parameters were balanced across the two cohorts, confirming adequate intergroup comparability (all *P* *>* *0.05*).

**Table 1:** Baseline demographic and anatomical characteristics of the two groups (mean ± standard deviation).

Variables	Experimental group (*n* = 133)	Control group (*n* = 133)	*t* value	*P value*
Age (years)	57.5 ± 15	57.6 ± 15.8	0.06	0.96
Height (m)	1.68 ± 0.07	1.68 ± 0.06	0.44	0.66
Body weight (kg)	65.37 ± 10.57	66.04 ± 10.54	0.54	0.59
BMI (kg/m^2^)	23.2 ± 2.87	23.38 ± 3.31	0.36	0.72
Maximum internal ring defect diameter (cm)	3.74 ± 0.47	3.72 ± 0.44	0.30	0.77

### 4.2 Operative time, intraoperative blood loss, postoperative 24 h pain score, and postoperative hospital stay

As shown in Table 2 operative duration was significantly longer in the experimental group (*P* < 0.05). No statistically significant intergroup differences were observed in intraoperative blood loss, 24 h postoperative VAS pain score, or postoperative hospital stay (all *P* *>* *0.05*).

**Table 2:** Perioperative outcome indicators (mean ± standard deviation).

Variables	Experimental group (*n* = 133)	Control group (*n* = 133)	*t* value	*P* value
Operative time (min)	56.84 ± 7.80	54.66 ± 7.88	2.27	0.02
Intraoperative blood loss (mL)	1.68 ± 0.07	1.68 ± 0.06	0.97	0.33
24 h postoperative VAS score	2.44 ± 1.32	2.38 ± 1.18	0.44	0.67
Postoperative hospital stay (d)	3.44 ± 1.34	3.21 ± 1.54	1.31	0.11

### 4.3 Postoperative seroma, recurrence and other complications

All 266 patients completed at least 6 months of follow-up; the median follow-up duration was 403 days, and the mean follow-up duration was 425.3 days. No surgical site infection, postoperative hemorrhage, chronic inguinal pain, spermatic cord entrapment, or nerve injury was recorded during follow-up.

As shown in Table 3 seroma developed in 15 patients (5.60%), including 3 cases (2.30%) in the experimental group and 12 cases (9.02%) in the control group. The intergroup difference was statistically significant (Fisher's exact test, *P* = 0.03). All seromas were identified within 1 week postoperatively via physical examination and subsequently confirmed by color Doppler ultrasound. In the experimental group, 1 seroma appeared on postoperative day 3 and resolved by the 2-week follow-up, while 2 seromas developed on postoperative day 7 and resolved at the 3-week follow-up. In the control group, 7 seromas appeared on postoperative day 3 and 5 on postoperative day 7; 10 resolved spontaneously within 2–3 weeks, 1 persisted until postoperative week 4 with complete ultrasound-confirmed resolution at week 5, and 1 required four ultrasound-guided percutaneous aspirations due to severe swelling, with full symptom relief at postoperative week 7. Regarding seroma classification, 1 Type II (0.60%) and 1 Type III (0.60%) seroma were observed in the control group, whereas no Type II or Type III seromas occurred in the experimental group. Owing to the low number of severe seroma events, statistical comparison between groups was not performed, and only descriptive analysis was provided.

**Table 3:** Comparison of postoperative recurrence and seroma incidence between the two groups [*n* (%)].

Group	*n*	Recurrence	Total seroma	Type I seroma	Type II seroma	Type III seroma	*P* value
Experimental group	133	0 (0)	3 (2.30)	3 (2.30)	0	0	–
Control group	133	2 (1.50)	12 (9.00)	10 (7.50)	1 (0.60)	1 (0.60)	–
*P* value		0.50^a^	0.03	–			–

a Only two recurrence events were observed, leading to severely limited statistical power. This *P* value is provided for descriptive reference only and cannot be used to infer intergroup differences in long-term recurrence risk.

Two recurrence events (0.75%) were recorded during follow-up, both limited to the control group. Given the relatively short median follow-up of 403 days, these data lack sufficient statistical power to evaluate intergroup differences in recurrence risk, and long-term follow-up is required for further validation.

## 5 Discussion

For Nyhus type III indirect inguinal hernias, the posterior abdominal wall presents a large defect with disrupted native supportive structures. Larger deep inguinal ring apertures and longer hernia sacs correspond to higher risks of postoperative seroma and recurrence. Even with intraoperative mesh coverage, large residual defects allow mesh migration or peritoneal bulging triggered by strenuous activity or coughing, which elevates recurrence risk.

Boldo E et al. (14) proposed that hernia sac volume, rather than mesh type or fixation technique, is the primary determinant of seroma formation. After hernia sac reduction, a dead space remains at the original hernia site. Surgical tissue irritation induces serous fluid leakage, and the potential space between implanted mesh and transversalis fascia allows fluid pooling to form seroma. Conventional surgical approaches cannot fully eliminate this dead space. Multiple investigators have tested diverse interventions to reduce these complications, yet no universal consensus has been established to date.

Intraoperative tissue dissection and transection by surgical instruments irritate the wound surface and exacerbate fluid accumulation. Theoretically, minimal tissue manipulation should reduce seroma incidence.

Li WM et al. (15) reported that hernia sac transection and fenestration reduced ultrasound-detected seroma volume, which they attributed to shortened use of thermal energy devices and decreased intraoperative thermal tissue injury. However, Sato et al. (16) conducted a larger retrospective study and found that reduced manipulation of the hernia sac and inguinal canal failed to lower seroma rates correspondingly. This finding indicates that seroma formation arises from the combined effects of multiple risk factors, and isolated reduction of tissue manipulation may not deliver clinical benefits. Against this background, the deep inguinal ring closure technique evaluated in this study acts through anatomical remodeling rather than merely minimizing tissue dissection to block fluid accumulation, yielding favorable clinical outcomes.

Ismail M et al. (17) placed closed-suction drains in the preperitoneal space during TEP surgery to evacuate fluid and gas, with drain removal at 24 h postoperatively. They reported that this strategy significantly reduced seroma incidence without increasing infection risk. Nevertheless, drain placement and removal add extra postoperative procedures, and the cutaneous drainage tract inevitably raises surgical site infection risk. Another trial assessed scrotal elevation as a physical intervention for laparoscopic/robotic inguinal hernia repair but confirmed no reduction in seroma formation (18), highlighting the need for alternative anti-seroma strategies. Notably, scrotal elevation significantly relieved early postoperative inguinal numbness and merits further large-scale investigation. The aforementioned interventions either increase surgical trauma or lack definitive efficacy, which motivated our exploration of a novel suturing technique that reinforces the posterior wall and obliterates residual dead space simultaneously.

In the present study, 3-0 absorbable barbed sutures were used laparoscopically for continuous suturing from the inferior margin of the transvs. abdominis arch above the deep inguinal ring down to the iliopubic tract below, fully closing the enlarged ring aperture. This maneuver restores the physiological caliber of the deep inguinal ring and improves mesh mechanical stability against intra-abdominal pressure. In addition, complete ring closure interrupts communication between the preperitoneal space and the dead space left by dissected hernia sacs, blocking fluid ingress into the residual cavity and decreasing postoperative seroma accumulation. In terms of safety and feasibility, although ring closure adds one extra surgical step, operative time was only approximately 2 min longer in the experimental group, with no clinically meaningful intergroup difference. Furthermore, postoperative VAS pain scores and hospital stay were comparable between groups, demonstrating that this suturing maneuver does not substantially increase surgical burden. This technique relies solely on cold suturing without additional use of thermal instruments, thereby avoiding extra serous leakage caused by thermal tissue injury. Importantly, indirect and direct hernias possess distinct anatomical substrates: direct hernia defects are located within Hesselbach's triangle and require repair of transversalis fascia defects, whereas indirect hernia repair targets closure of the deep inguinal ring and surrounding fascial tissues. Based on these mechanistic differences, we further compared postoperative seroma incidence between the two study groups.

All patients received routine physical examination during outpatient follow-up, while ultrasound was not performed universally. This protocol was adopted for the following reasons: small ultrasound-only fluid collections without palpable swelling are typically asymptomatic, self-limiting, and rarely cause pain or secondary complications. Universal postoperative ultrasound would substantially increase medical costs and impose unnecessary psychological stress on patients. Accordingly, ultrasound was only ordered selectively for patients with palpable swelling or clinical suspicion of seroma. In this cohort, all clinically palpable seromas were confirmed via color Doppler ultrasound; minor collections resolved spontaneously within 2–3 weeks under observation, one larger collection persisted until week 5, and one severely symptomatic seroma resolved after repeated percutaneous aspiration. No abnormal physical findings were identified in the remaining patients, and their follow-up courses were uncomplicated.

This study demonstrated that deep inguinal ring closure reduced the incidence of clinically symptomatic seroma from 9.02% to 2.30%, consistent with the mechanistic pathways described above. The precise molecular and anatomical mechanisms by which ring closure suppresses seroma development require further validation via dedicated imaging studies. Consistent with existing evidence supporting defect closure for direct hernias, our results confirmed protective effects of deep ring closure for indirect hernias, suggesting that fascial ring closure may be applicable across hernia subtypes. However, individualized adjustment of suturing techniques and safety margins is required according to patient-specific inguinal anatomy.

This study carries several limitations. Although baseline variables were well balanced between groups, the retrospective single-center design introduces potential unmeasured confounders (e.g., hernia sac volume, degree of scrotal extension) that may bias outcomes. Enrollment was restricted to adult male patients with Nyhus type III indirect hernias, limiting the generalizability of our conclusions. In addition, the median follow-up of only 403 days is insufficient to evaluate long-term recurrence risk, and merely two recurrence events were observed, resulting in inadequate statistical power to draw reliable conclusions regarding long-term recurrence rates; prospective long-term follow-up is required for validation. Finally, ultrasound was only performed for patients with positive physical examination findings to confirm seroma, so the reported incidence reflects clinically significant seromas rather than the total rate of radiologically detected asymptomatic fluid collections.

## 6 Conclusion

For adult male patients with Nyhus type III indirect inguinal hernia undergoing TAPP repair, sutured closure of the deep inguinal ring using absorbable barbed sutures is safe and feasible. This technique only slightly prolongs operative time without increasing surgical complications and reduces the incidence of early postoperative symptomatic seroma. Prospective randomized controlled trials with larger sample sizes and extended long-term follow-up are required to verify its sustained impact on hernia recurrence rates.

## Data Availability

The original contributions presented in the study are included in the article/Supplementary Material, further inquiries can be directed to the corresponding authors.
